# Exendin-4 in combination with adipose-derived stem cells promotes angiogenesis and improves diabetic wound healing

**DOI:** 10.1186/s12967-017-1145-4

**Published:** 2017-02-15

**Authors:** Eunhui Seo, Jae Soo Lim, Jin-Bum Jun, Woohyuk Choi, In-Sun Hong, Hee-Sook Jun

**Affiliations:** 1 College of Pharmacy and Gachon Institute of Pharmaceutical Science, Gachon University, Incheon, 21936 Republic of Korea; 2 Lee Gil Ya Cancer and Diabetes Institute, Gachon University, Incheon, 21999 Republic of Korea; 3 Division of Life Sciences, Korea University, Seoul, 02841 Republic of Korea; 4 Gachon Medical Research Institute, Gil Hospital, Incheon, 21565 Republic of Korea

**Keywords:** Diabetic wound, Angiogenesis, Exendin-4, GLP-1, Adipose-derived stem cells

## Abstract

**Background:**

Diminished wound healing is a major complication of diabetes mellitus and can lead to foot ulcers. However, there are limited therapeutic methods to treat this condition. Exendin-4 (Ex-4), a glucagon-like peptide-1 receptor agonist, is known to have many beneficial effects on diabetes. In addition, mesenchymal stem cells are known to have wound healing effects. We investigated the effects of Ex-4 in combination with human adipose tissue-derived stem cells (ADSCs) on diabetic wound healing in a diabetic animal model.

**Methods:**

Diabetic *db/db* (blood glucose levels, >500 mg/dl) or C57BL/6 mice were subjected to wounding on the skin of the back. One day after wounding, each wound received ADSCs (2.5 × 10^5^ cells) injected intradermally around the wound and/or Ex-4 (50 μl of 100 nM Ex-4) topically applied on the wound with a fine brush daily. Wound size was monitored and wound histology was examined. Human endothelial cells and keratinocyte cells were used to assess angiogenesis and vascular endothelial growth factor expression in vitro.

**Results:**

Topical administration of Ex-4 or injection of ADSCs resulted in a rapid reduction of wound size in both diabetic and normoglycemic animals compared with vehicle treatment. Histological analysis also showed rapid skin reconstruction in Ex-4-treated or ADSC-injected wounds. A combination of Ex-4 and ADSCs showed a significantly better therapeutic effect over either treatment alone. In vitro angiogenesis assays showed that both Ex-4 and ADSC-conditioned media (CM) treatment improved migration, invasion and proliferation of human endothelial cells. ADSC-CM also increased migration and proliferation of human keratinocytes. In addition, both Ex-4 and ADSC-CM increased the expression of vascular endothelial growth factor. Co-culture with ADSCs increased migration and proliferation of these cells similar to that found after ADSC-CM treatment.

**Conclusions:**

We suggest that Ex-4 itself is effective for the treatment of diabetic skin wounds, and a combination of topical treatment of Ex-4 and injection of ADSCs has a better therapeutic effect. Thus, a combination of Ex-4 and ADSCs might be an effective therapeutic option for the treatment of diabetic wounds, such as foot ulcers.

## Background

Diabetes mellitus is a metabolic disease, and the incidence of diabetes is escalating everywhere in the world [1]. The disease often leads to the development of serious complications such as diabetic retinopathy, nephropathy, neuropathy, and vascular complications [2]. Diabetic foot ulcers, which are caused by diminished wound healing, are one of the most serious and costly complications [2, 3] and a major risk factor for lower-limb amputation [4]. New research on advanced therapeutic products such as stem cells, growth factors, skin substitutes, and gene therapy has been conducted. Despite these new therapeutic methods, their efficacy remains poor. More effective therapeutic methods are still needed.

Angiogenesis is a critical component of wound healing [5], and the development of chronic wounds in diabetes are mainly due to the reduction of angiogenic ability [6]. Therefore, one of the main aims of diabetic wound healing therapy includes improving angiogenesis. The proliferation and migration of keratinocytes, called re-epithelialization, is also critical to wound healing [7]. Both proliferation and migration of keratinocytes are stimulated by the local wound area and keratinocyte-produced cytokines such as vascular endothelial growth factor (VEGF) and insulin-like growth factor-1 [7, 8].

Several studies have indicated that mesenchymal stem cells migrate to the wound site during wound healing [9, 10], and it is well known that adipose tissue-derived stem cells (ADSCs) and secretory factors from ADSCs promote wound healing [11, 12]. However, stem cell therapy is costly and cell survival rate is very low. Another strategy to improve wound healing is treatment with growth factors such as VEGF, fibroblast growth factor and platelet-derived growth factor [13, 14].

Glucagon-like peptide-1 (GLP-1), an incretin hormone secreted from enteroendocrine L cells, is known to have many anti-diabetic effects [15]. Exendin-4 (Ex-4), a GLP-1 receptor agonist with a longer half-life than GLP-1, has been developed for the treatment of type 2 diabetes mellitus [16]. GLP-1 and Ex-4 also have anti-oxidative and anti-inflammatory effects [17, 18], which might be beneficial for the wound healing process.

In this study we investigated the effect of a combination of Ex-4 and ADSCs on experimental skin wounds in diabetic mice. Our data show that Ex-4 itself is effective for the treatment of diabetic skin wounds, and a combination of Ex-4 and ADSCs showed better therapeutic effects on diabetic wound healing.

## Methods

### Cell culture

Human umbilical vein endothelial cells (HUVECs) and human keratinocytes (HaCaT cells) were obtained from ATCC (Manassas, VA). The cells were cultured according to the instructions from the supplier. HUVECs were used between passage 4 and passage 8 for experiments. Human adipose-derived stem cells (ADSCs) were obtained from Invitrogen (Carlsbad, CA, USA) and cultured in MesenPRO RS^TM^ medium (Invitrogen, Carlsbad, CA, USA).

### Animal procedures

Db/db mice were supplied by the Korea Research Institute of Bioscience and Biotechnology, (Daejeon, Korea). C57BL/6 mice were supplied by Orient Bio (Sungnam, Korea). Diabetic db/db mice (blood glucose >500 mg/dl) or normoglycemic C57BL/6 mice (8 week-old males) were divided into five groups: control (no wound); wound + vehicle; wound + Ex-4; wound + ADSCs; wound + ADSCs + Ex (n = 7–10, each group). Animals were subjected to wounding (6 mm in diameter) by punch biopsy (Stiefel, Bad Oldesloe, Germany) on the skin of the back of the mouse. Prior to wounding, general anesthesia was induced by the administration of isoflurane. The hair was shaved and the skin was sterilized with povidone–iodine solution. After wounding, mice were maintained in separate cages and given access to food and water ad libitum. One day after wounding, mice in the experimental groups received 2.5 × 10^5^ ADSCs in phosphate-buffered saline (PBS) injected intradermally around the wound and/or 50 μl of 100 nM Ex-4 (Sigma-Aldrich, St. Louis, MO) in 0.5% collagen-PBS applied to the wound with a fine brush daily. Mice in the vehicle-treated group were treated with 0.5% collagen-PBS daily. To assess the rate of wound healing, images of all wounds were recorded using a digital camera at the time of wounding (day 0) and on a daily basis post-wounding. The wound areas were determined on photographs using Adobe Photoshop CS5.1 program (Adobe, San Jose, Calif.)

### Wound histology

On postoperative day 14, the animals were sacrificed and the wounded tissues and surrounding skins were removed. The tissues were fixed in 10% neutral buffered formalin, embedded in paraffin and sectioned. The sections were stained with hematoxylin/eosin (H&E) and Masson’s trichrome. For H&E staining, slides were deparaffinized by incubation in xylene, hydrated by washes in a series of ethanol (100, 95, 80, and 70%), washed in distilled water, and stained with H&E (Sigma-Aldrich). Dermal thickness was determined by measuring the distance between the epidermal–dermal junction and the dermal–subcutaneous-fat junction in the center of recovered wound area using Adobe Photoshop CS5.1 program. Masson’s trichrome staining was performed using the IHC World NovaUltra Masson Trichrome stain kit (Woodtsock, MD) as described by the manufacturer. Collagen densities, which were visualized with Masson-trichrome staining, were measured with a histogram created with Adobe Photoshop CS5.1 program. For VEGF staining, the sections were incubated for 16 h with rabbit polyclonal antibodies against VEGF (Santa Cruz, CA, USA) diluted 1:100 in 0.1% PBS containing 0.3% TritonX-100 at 4 °C. After washing, the sections were then incubated with FITC-conjugated secondary antibodies for 1 h at room temperature and counterstained with 4′,6-diamidino-2-phenylindole (DAPI). The VEGF stained areas were measured with a histogram created with Adobe Photoshop CS5.1 program.

### Measurement of hemoglobin A1C (HbA1c) levels and blood glucose levels

On day 14 after wounding, blood samples were obtained from the tail vein. Hemoglobin A1c (HbA1c) measurements were made using an AU 680 chemistry analyzer (Beckman Coulter, Inc. Brea, CA) and an HbA1c APT kit (Beckman Coulter, Inc.) following the manufacturer’s instructions. HbA1c <6% was considered normal [19]. Blood glucose levels were measured with a glucose analyzer (OneTouch^®^ Ultra, Lifescan, Johnson & Johnson, Milpitas, CA).

### Wounding migration assay

HUVECs or HaCaT cells were plated on 24-well plates (Nunc, Rochester, NY, USA) and grown to 90% confluence. Cells were wounded with a 1-mm-wide pipette tip, and the injury line was marked. After wounding, the cultures were washed with serum-free medium and incubated with or without 10 nM Ex-4 and/or ADSC-conditioned media (CM) to evaluate the effect on the ADSC CM. ADSC-CM was prepared by incubating ADSCs with serum-free DMEM for 24 h. To investigate whether co-culture with ADSCs would have a similar effect to ADSC-CM, transwell plates (24-well plate, Corning Costar, Cambridge, MA) were used and ADSCs were seeded in the upper chamber (5 × 10^4^ cells/well). Cells undergoing migration assay were seeded in the lower compartment. Cells were allowed to migrate for 24 h and then rinsed with serum-free medium. Migration patterns were observed under a phase contrast microscope and photographed.

### Invasion assay

The invasion capacity of HUVECs was determined using a modified 24-well Boyden chamber (8 μm-pore size) (Corning Incorporated, Corning, NY). For the invasion measurements, the filter membrane was coated with 1 mg/ml Matrigel (BD Biosciences, Franklin Lakes, NJ) using 20 μl volume per well. The cells were seeded at a density of 2 × 10^4^ cells in 100 μl medium in the upper compartment of the transwell and incubated with or without 10 nM Ex-4 and/or ADSC-CM. After 24 h of incubation at 37 °C, cells that did not penetrate the filter were wiped off with cotton swabs, and cells that had migrated to the lower surface of the filter were fixed with methanol. Fixed cells were then stained with crystal violet, observed under a phase contrast microscope and photographed.

### Proliferation assay

Cells were plated on 96-well plates and incubated with or without ADSC-CM and/or 10 nM Ex-4. To investigate whether co-culture with ADSCs would have a similar effect to ADSC-CM, transwell plates (24-well plate, Corning Costar, Cambridge, MA) were used and ADSCs were seeded in the upper chamber (5 × 10^4^ cells/well). A Cell Counting Kit-8 (CCK-8) (Dojindo Laboratories, Kumamoto, Japan) was used to measure cell viability.

### Western blotting

Cells were homogenized with Mammalian Protein Extraction Buffer (GE Healthcare, Milwaukee, WI) containing a protease and phosphatase inhibitor cocktail (Sigma-Aldrich). The total proteins (30 μg) were resolved by 12% sodium dodecyl sulfate polyacrylamide gel electrophoresis, transferred onto membranes, and blocked with tris buffered saline containing Tween 20 in 5% non-fat dry milk. The membranes were incubated with anti-VEGF and anti- β-actin antibodies (Santa Cruz, CA, USA) and visualized by incubating with horseradish peroxidase-conjugated secondary antibodies (Santa Cruz) followed by Immobilon Western Chemiluminescent HRP Substrate (Millipore, St. Charles, MO). Chemiluminescence was detected by LAS-4000 (Fuji Film, Tokyo, Japan). The images derived from western blotting were analyzed through ImageJ (National Institutes of Health, Bethesda, MD) software for Windows.

### Statistical Analyses

All data are expressed as mean ± standard error of at least three independent experiments. Data were analyzed using Analysis of Variance followed by post hoc analysis using the Tukey range test (SPSS 10.0 statistical software). p values less than 0.05 were considered statistically significant.

## Results and discussion

### Combination of Ex-4 and ADSC treatment showed synergistic effect on wound healing in diabetic db/db mice

**Fig. 1:**
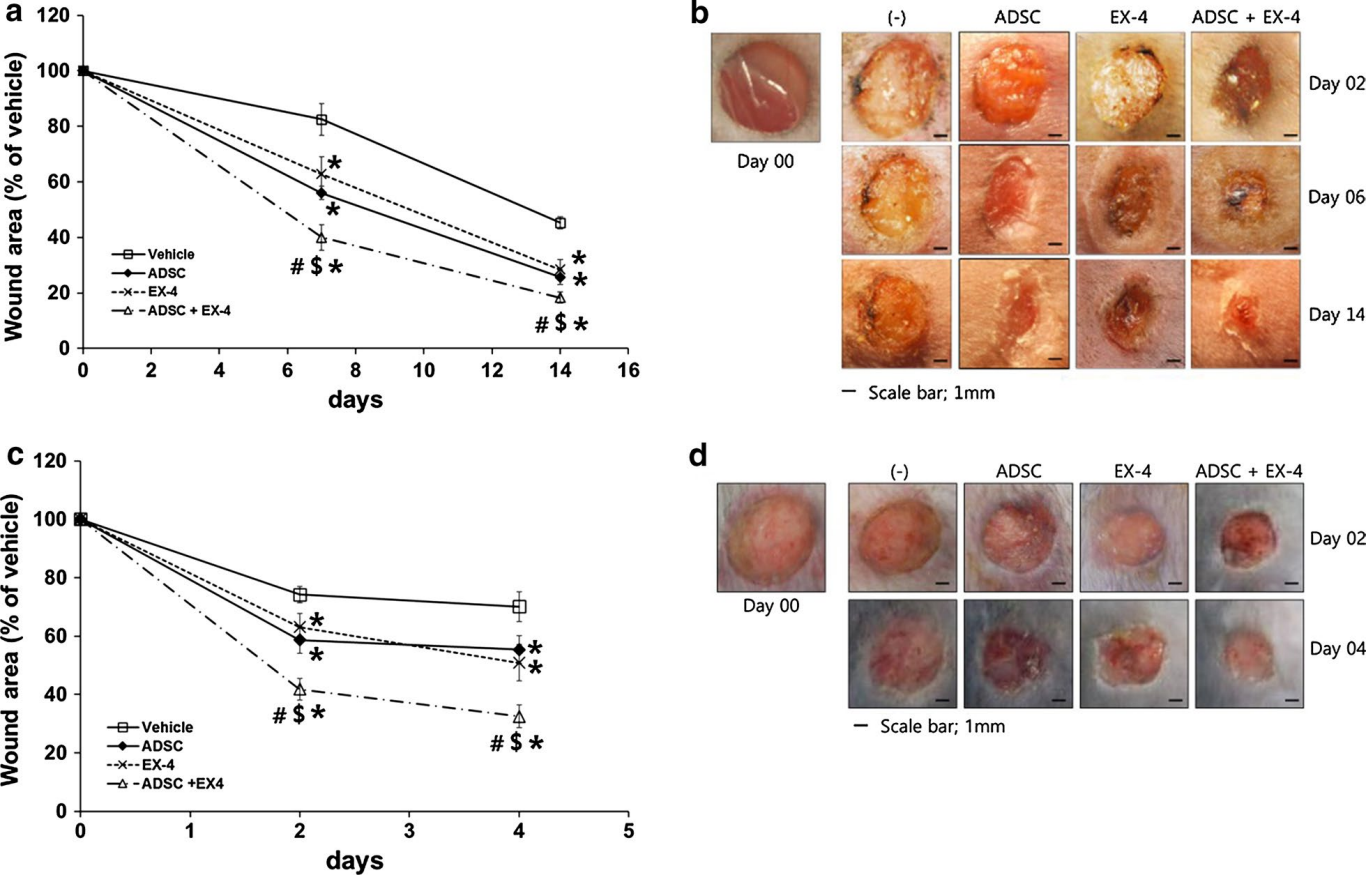
Effect of Ex-4, ADSCs, or a combination of Ex-4 and ADSCs on diabetic wound healing. Diabetic db/db mice (**a**, **b**) or normal C57BL/6 mice (**c**, **d**) were subjected to wounding (6 mm in diameter) by punch biopsy on the skin of the back. One day later, each wound received 2.5 × 10^5^ ADSCs injected intradermally around the wound and/or 50 μl of 100 nM Ex-4 was applied to the wound with a fine brush, daily. **a** Changes of wound area at each of the time points compared with the original wound area. Values represent mean ± SEM. *p < 0.05 vs vehicle, ^$^ *p* < 0.05 vs ADSC, ^#^ *p* < 0.05 vs EX-4. **b** Wound shapes were recorded over 14 days. **c** Wound area up to 4 days after artificial wound creation. *p < 0.05 vs vehicle, ^$^ *p* < 0.05 vs ADSC, ^#^ *p* < 0.05 vs EX-4. **d** Wound shapes 2 and 4 days after artificial wound creation

ADSCs are a promising stem cell source for regenerative medicine and wound repair [11, 20, 21]. Ex-4 is known to induce proliferation of endothelial cells and promote angiogenic effects [22], and Ex-4 treatment prevents hindlimb ischemic injury [23]. For these reasons, we investigated the effects of Ex-4 and ADSCs on wound repair in diabetic mice. Each mouse sustained an artificial wound given with a sterile punch biopsy tool in a surgical procedure. Topical treatment with Ex-4 or ADSC injection healed the wound faster compared with vehicle treatment in diabetic mice. There were no significant differences between Ex-4 treatment and ADSC transplantation in wound healing rate. However, the combination of Ex-4 and ADSCs showed a significantly better therapeutic effect over the single treatment (14 days after wound creation, p = 0.016 vs ADSC, p = 0.012 vs EX-4) (Fig. 1a, b). When we examined wound healing in normoglycemic C57BL/6 mice, Ex-4 treatment or ADSC transplantation accelerated wound healing and were more effective when combined (Fig. 1c, d).

**Fig. 2:**
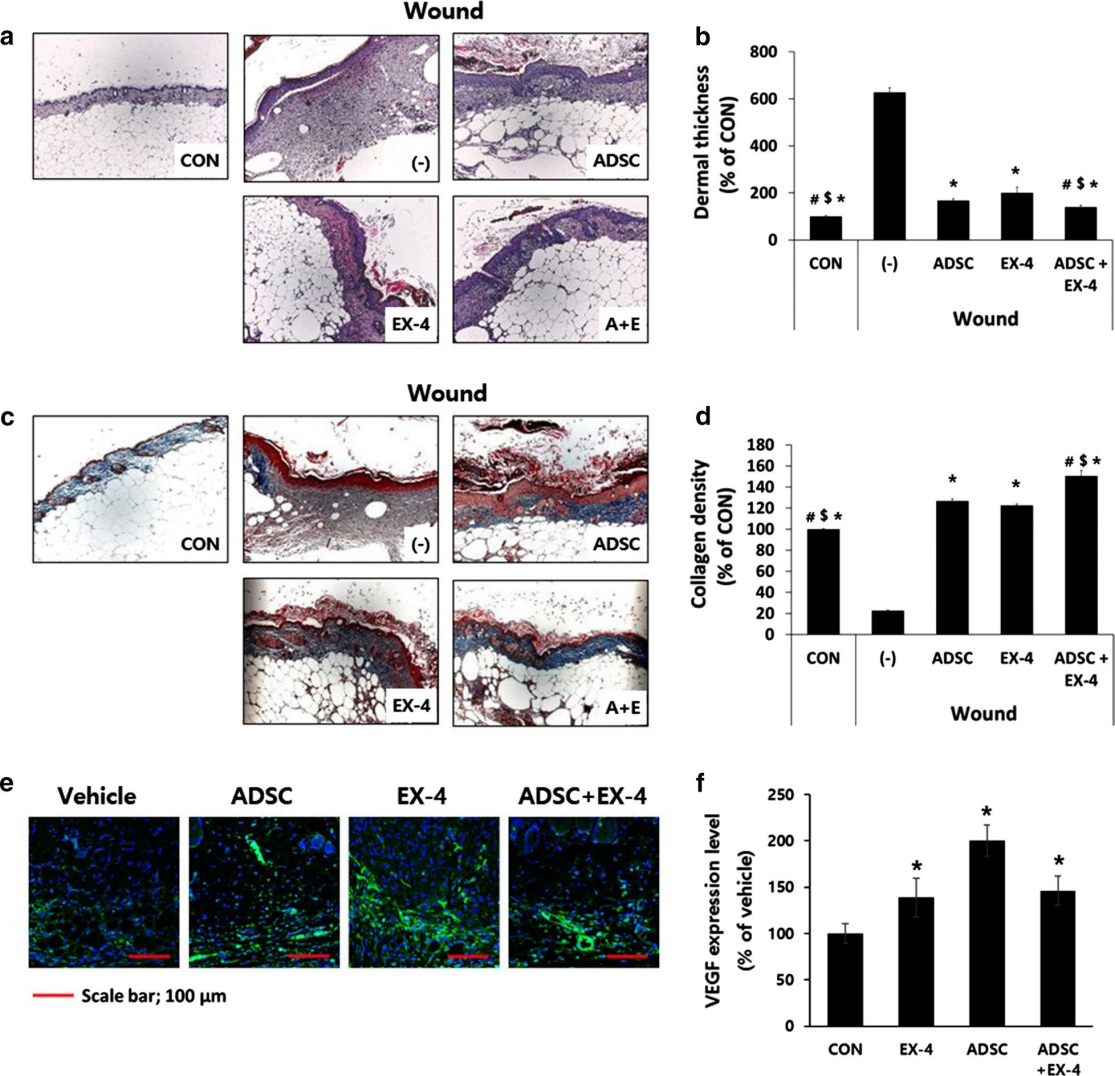
Histological analysis of skin morphology of wounds on day 14 after treatment with Ex-4, ADSCs, or combination of Ex-4 and ADSCs. Diabetic db/db mice were subjected to wounding (6 mm in diameter) by punch biopsy on the skin of the back. One day later, each wound received 2.5 × 10^5^ ADSCs injected intradermally around the wound and/or 100 nM Ex-4 was applied to the wound with a fine brush, daily. **a** H&E staining of wound area (×400). **b** The thickness of the dermis observed through the H & E staining results. *p < 0.05 vs wound-(-), ^$^ *p* < 0.05 vs wound-ADSC, ^#^ *p* < 0.05 vs wound-EX-4. **c** Masson’s trichrome staining of wound area (×400). **c** Masson’s trichrome staining of wound area (×400). **d** The collagen density observed through Masson’s trichrome staining results. *p < 0.05 vs wound-(−), ^$^ *p* < 0.05 vs wound-ADSC, ^#^ *p* < 0.05 vs wound-EX-4. **e** Immunofluorescence staining of VEGF. (×800). **f** VEGF expression levels observed through VEGF staining results. *p < 0.05 vs vehicle, ^#^ *p* < 0.05 vs EX-4

Histological analysis of the wounds on day 14 after wounding showed that the epidermis and dermis in Ex-4-treated and/or ADSC transplanted wounds were more re-epithelialized, normalized and closed than in vehicle-treated wounds (Fig. 2a). The thickness of the dermis was also reduced by Ex-4 treatment or ADSC transplantation compared to the vehicle treatment and further reduced by combination treatment of Ex-4 and ADSCs (Fig. 2b). This is likely due to the infiltration of fibroblasts and myofibroblasts, contributing to tissue turnover and reorganization. Masson’s trichrome staining and quantitative analysis of the stained area demonstrated that Ex-4 treatment and ADSC transplantation markedly reduced collagen deposition compared with vehicle treatment (Fig. 2c, d). Similarly, another study showed that intradermal injection of Ex-4 promoted wound healing on abraded skin in normoglycemic mice [24]. Like the wound healing rate, we found no significant differences between Ex-4 treatment and ADSC transplantation in histological assays. But, the combination of ADSC and Ex-4 treatment showed more complete re-epithelialization than that seen with either ADSCs or Ex-4 alone. This observation suggests that ADSCs and Ex-4 not only increased the rate of healing, but also increased the normalization of the wound through more effective re-epithelialization.

It is well known that chronic wounds in diabetes are mainly the result of lack of angiogenesis [6]. Angiogenesis occurs by activating endothelial cells and smooth muscle cells, and by triggering cell migration, invasion, proliferation, and formation of tubular structures [27]. VEGF is an important angiogenic factor in wound healing [25], Immunofluorescence staining of VEGF expression in wounded tissues revealed that VEGF expression was higher in all treatment groups than in the vehicle group (Fig. 2e, f). These results suggest that the increased expression of VEGF might contribute to the acceleration of wound healing.

### Topical Ex-4, ADSCs, or combination of Ex-4 and ADSC treatment did not affect glucose homeostasis in db/db mice

**Fig. 3:**
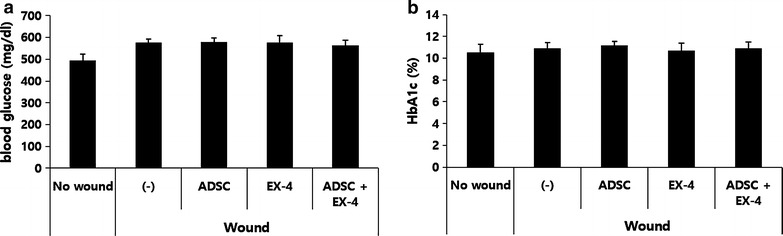
The effect of treatment with Ex-4, ADSCs, or combination of Ex-4 and ADSCs on diabetes. **a**, **b** Diabetic db/db mice were subjected to wounding (6 mm in diameter) by punch biopsy on the skin of the back. One day later, each wound received 2.5 × 10^5^ ADSCs injected intradermally around the wound and/or 100 nM Ex-4 was applied on the wound with a fine brush, daily. Fourteen days after wounding, **a** blood glucose and **b**. HbA1c levels were measured

Because hyperglycemia is the main cause of the reduction of angiogenic ability and wound healing [6], we investigated whether topical administration of Ex-4 or local injection of ADSCs into diabetic wounds show an effect on hyperglycemia. At 14 days after wounding, blood glucose and HbA1c levels were measured. There were no differences among any of the groups (Fig. 3). Although Ex-4 is well known to have blood glucose lowering effects in diabetes when given systemically [26, 27], topical administration of Ex-4 to the artificial wounds had no effect on blood glucose levels in our study. These results show that the wound healing effect of Ex-4 and ADSCs is not caused by a reduction of blood glucose levels and improvement of diabetes, but rather that Ex-4 and ADSCs have direct wound healing effects.

### Ex-4 or ADSC-CM have angiogenic effects in human endothelial cells

**Fig. 4:**
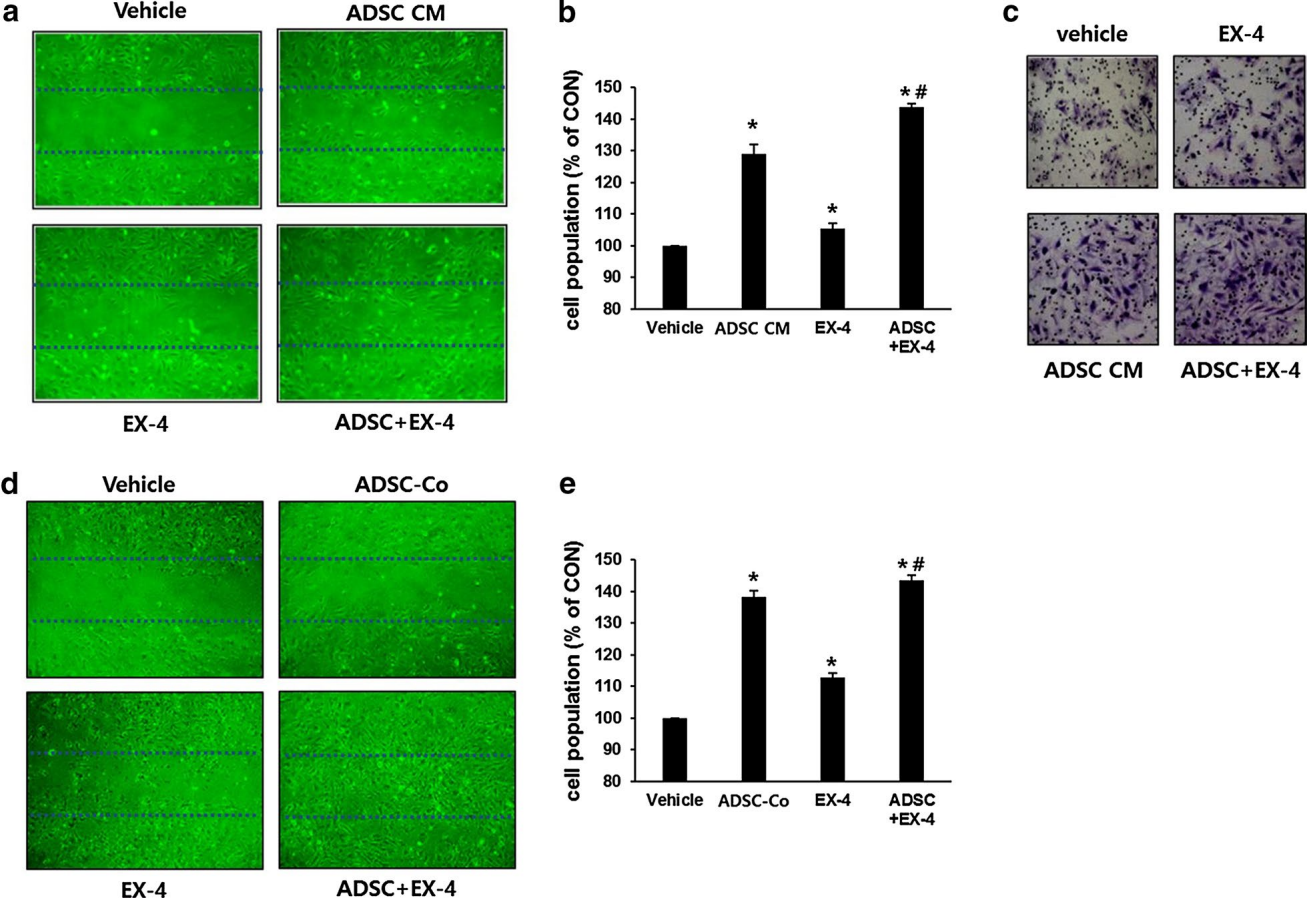
The effects of Ex-4 and ADSCs on migration, proliferation and invasion of HUVECs. **a**, **d** Migration assay. Plated HUVECs were wounded and the injury line was marked. **a** Cells were cultured with Ex-4 and/or ADSC-CM. **d** Cells were co-cultured with or without ADCSs in the *upper chamber* of a transwell plate with or without Ex-4 treatment. Migration patterns were observed under a phase contrast microscope and photographed (×200). **b**, **e** Proliferation assay. **b** HUVECs were plated and incubated with or without ADSC-CM and/or 10 nM Ex-4. **e** HUVECs were plated on transwell plates and co-cultured with ADSCs (in the *upper chamber*) and/or 10 nM Ex-4. After 24 h, cell proliferation was determined with a cell counting Kit-8 assay. Results are expressed as a percentage of the control. *p < 0.05 vs CON, ^#^ *p* < 0.05 vs ADSC. **c** Invasion assay. HUVECs (2 × 10^4^ cells) were seeded into the upper compartment of a transwell and incubated with or without ADSC-CM and/or 10 nM Ex-4 for 24 h. Sample images are shown of transmigrated cells stained with crystal violet (×200)

To test whether Ex-4 and ADSCs has angiogenic effects, cell migration assays were carried out. In vitro scratch wound assays using HUVECs found that Ex-4 or ADSC-CM treatment enhanced the migration of endothelial cells compared with vehicle treatment, and a combination of Ex-4 and ADSC-CM showed a significantly better effect than either treatment alone (Fig. 4a). Proliferation assays showed that proliferation was significantly increased after treatment with Ex-4, or ADSC-CM, and the combination of both produced the best effect (Fig. 4b). Invasion assays using HUVECs revealed that Ex-4 or ADSC-CM treatment resulted in better invasion compared with the control, that ADSC-CM appeared to have a better effect than Ex-4, and that a combination of both showed the best effect (Fig. 4c).

Similarly, ADSC co-culture showed enhanced the migration of endothelial cells (Fig. 4d) and proliferation (Fig. 4e) as found using ADSC-CM. It was reported that co-culture of endothelial cells and ADSCs induce vascular tube formation by outgrowth endothelial cells [28] and that secretory factors from ADSCs have a large effect on wound healing [11]. In our study, we found that ADSC-CM and the co-culture system showed similar effects on proliferation.

### ADSC-CM, but not Ex-4, induced the migration and proliferation of human keratinocytes

**Fig. 5:**
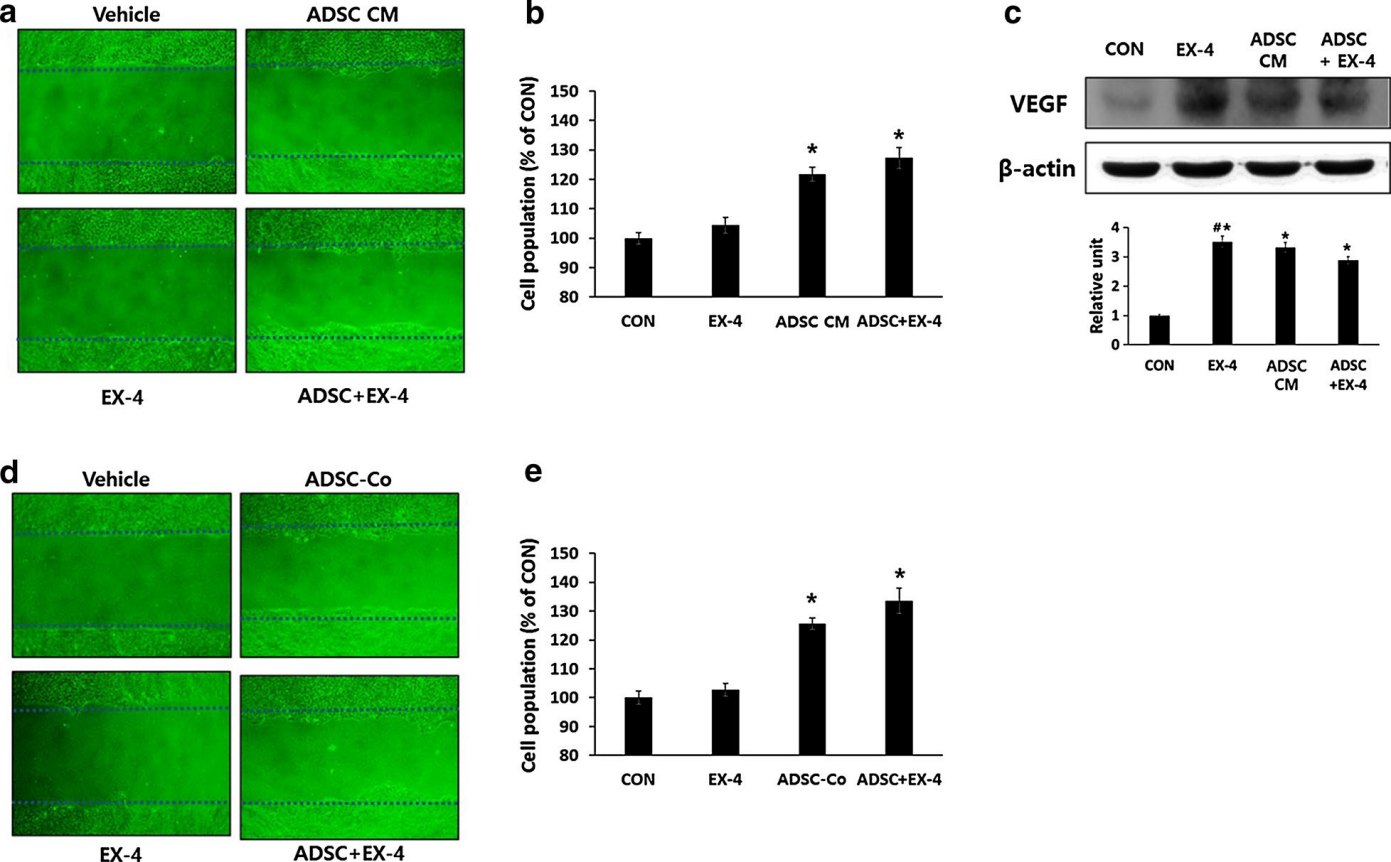
The effect Ex-4 or ADSC-CM on migration, proliferation and angiogenic factor secretion in keratinocytes. **a**, **d** Migration assay. Plated HaCaT cells were wounded and the injury line was marked. **a** Cells were cultured with ADSC-CM and/or 10 nM Ex-4. **d** Cells were co-cultured with or without ADCSs in the upper chamber of a transwell plate with or without Ex-4 treatment. Migration patterns were observed under a phase contrast microscope and photographed (×200). **b**, **e** Proliferation assay. **b** HaCaT cells were plated on 96-well plates and incubated with or without ADSC-CM and/or 10 nM Ex-4. **e** HaCaT cells were plated on transwell plates and co-cultured with ADSCs (in the *upper chamber*) and/or 10 nM Ex-4. After 24 h, cell proliferation was determined with a cell counting Kit-8 assay. Results are expressed as a percentage of the control. *p < 0.05 vs CON. **c** Western blotting assay. HaCaT cells were incubated with or without ADSC-CM and/or 10 nM Ex-4. After 24 h, cells were harvested and western blotting assay for VEGF was carried out. The results were analyzed through ImageJ software for Windows. *p < 0.05 vs CON, ^#^ *p* < 0.05 vs ADSC + EX-4

In the course of wound healing, keratinocytes migrate from the basal population in the region of the wound boundary to cover the damage and repair the barrier function of the skin [29]. To investigate the effects of Ex-4 or ADSC-CM treatment on human keratinocyte migration, a migration assay was carried out using HaCaT cells, a human keratinocyte cell line. ADSC-CM treatment stimulated keratinocyte migration, whereas Ex-4 treatment had no effect (Fig. 5a). The results of ADSCs co-culture system were parallel to those of ADSC-CM (Fig. 5d) Similarly, ADSC-CM treatment or ADSC co-culture, but not Ex-4 treatment, induced keratinocyte proliferation (Fig. 5b, e). These results suggest that migration and proliferation of keratinocytes is stimulated by ADSC-CM rather than Ex-4.

### Both Ex-4 and ADSCs CM increased VEGF expression in human keratinocytes

VEGF expression was increased in wound area treated with Ex-4 or ADSCs or both (Fig. 2e, f) and it is well known that keratinocytes are a source of VEGF [8]. We found that treatment with either ADSC-CM or Ex-4 stimulated VEGF expression in human keratinocytes, and combination treatment did not further increase the expression of VEGF (Fig. 5c). Several studies have reported that GLP-1 or GLP-1 analogs stimuate VEGF expression in various cells [30–32]. In a mouse hindlimb ischemia model, Ex-4 prevents injury and increases VEGF [23]. As well, Raffaele et al. reported that vildagliptin, which reduces the rapid degredation of GLP-1, improves diabetic wounds of type 2 diabetic patients by improving VEGF generation and upregulating of hypoxia-inducible factor 1α in wound specimens [33]. Our results are consistent with these findings.

## Conclusions

In this study, we demonstrated that either topical Ex-4 treatment or local injection of ADSCs are effective for the treatment of experimental skin wounds in diabetic db/db mice, however a combination of Ex-4 and ADSCs have the best therapeutic effect. We found that Ex-4 has angiogenic effects on endothelial cells, whereas ADSCs have angiogenic effects on both endothelial cells and keratinocytes, contributing to the acceleration of re-epithelization and wound closure. Determining whether or not the beneficial effects of Ex-4 and ADSCs on diabetic wound healing are additive or synergistic will require further study.

## Abbreviations

ADSCs: human adipose-derived stem cells
ADSC-CM: human adipose-derived stem cell-conditioned media
Ex-4: exendin-4
GLP-1: glucagon-like peptide-1
HbA1c: hemoglobin A1c
H&E: hematoxylin and eosin
HUVECs: human umbilical vein endothelial cells
PBS: phosphate-buffered saline
VEGF: vascular endothelial growth factor
